# *C9ORF72*-related cellular pathology in skeletal myocytes derived from ALS-patient induced pluripotent stem cells

**DOI:** 10.1242/dmm.039552

**Published:** 2019-08-16

**Authors:** Eileen Lynch, Theran Semrad, Vincent S. Belsito, Claire FitzGibbons, Megan Reilly, Koji Hayakawa, Masatoshi Suzuki

**Affiliations:** 1 Department of Comparative Biosciences, University of Wisconsin-Madison, Madison, WI 53706, USA; 2 Department of Toxicology, Faculty of Veterinary Medicine, Okayama University of Science, Imabari, Ehime 794-8555, Japan; 3 Stem Cell and Regenerative Medicine Center, University of Wisconsin-Madison, Madison, WI 53706, USA

**Keywords:** iPSCs, Skeletal muscle, TDP-43, Amyotrophic lateral sclerosis

## Abstract

Amyotrophic lateral sclerosis (ALS) is a late-onset neuromuscular disease with no cure and limited treatment options. Patients experience a gradual paralysis leading to death from respiratory complications on average only 2-5 years after diagnosis. There is increasing evidence that skeletal muscle is affected early in the disease process, yet the pathological processes occurring in the skeletal muscle of ALS patients are still mostly unknown. Specifically, the most common genetic cause of ALS, a hexanucleotide repeat expansion in the *C9ORF72* gene, has yet to be fully characterized in the context of skeletal muscle. In this study, we used the protocol previously developed in our lab to differentiate skeletal myocytes from induced pluripotent stem cells (iPSCs) of *C9ORF72* ALS (C9-ALS) patients in order to create an *in vitro* disease model of C9-ALS skeletal muscle pathology. Of the three *C9ORF72* mutation hallmarks, we did not see any evidence of haploinsufficiency, but we did detect RNA foci and dipeptide repeat (DPR) proteins. Additional abnormalities included changes in the expression of mitochondrial genes and a susceptibility to oxidative stress, indicating that mitochondrial dysfunction may be a critical feature of C9-ALS skeletal muscle pathology. Finally, the C9-ALS myocytes had increased expression and aggregation of TDP-43. Together, these data show that skeletal muscle cells experience pathological changes due to the *C9ORF72* mutation. Our *in vitro* model could facilitate further study of cellular and molecular pathology in ALS skeletal muscle in order to discover new therapeutic targets against this devastating disease.

This article has an associated First Person interview with the first author of the paper.

## Abstract (precis)

**Summary:** Evidence of protein aggregation and mitochondrial dysfunction were found in skeletal myocytes differentiated from ALS-patient induced pluripotent stem cells with the *C9ORF72* mutation.

## INTRODUCTION

Amyotrophic lateral sclerosis (ALS) is a deadly neuromuscular disease featuring motor neuron cell death and skeletal muscle atrophy and wasting leading to gradual loss of motor function (Brown and Al-Chalabi, 2017; Loeffler et al., 2016). The disease typically has a late onset of symptoms but a quick progression, with death from respiratory failure occurring on average 2-5 years after diagnosis. About 90% of ALS cases are considered sporadic, with only 10% accounted for by genetically inherited mutations. There are around 25 different mutations that have been implicated to cause either sporadic or familial ALS, or both (Brown and Al-Chalabi, 2017). Some of the most commonly studied mutations include *SOD1*, *TARDBP*, *FUS* and, more recently, *C9ORF72*. Together, these four genes are responsible for greater than 50% of familial ALS cases (Corcia et al., 2017). Many of the proteins encoded by genes mutated in ALS patients are involved in protein homeostasis, RNA processing and cytoskeletal organization (Brown and Al-Chalabi, 2017; Vucic et al., 2014), as well as mitochondrial function (Carrì et al., 2017; Khalil and Liévens, 2017). Consequently, the motor neurons of ALS patients have been shown to experience protein aggregation, mitochondrial dysfunction, oxidative stress, defective axonal transport and excitotoxicity, ultimately leading to motor neuron degeneration (Ferraiuolo et al., 2011).

The hexanucleotide GGGGCC repeat expansion in the open reading frame of chromosome 9 (*C9ORF72*) is the most common cause of familial ALS and is also found in many sporadic cases (DeJesus-Hernandez et al., 2011; Renton et al., 2011). There are three proposed mechanisms for how the repeat expansion may result in cellular pathology. The first is a loss of function caused by haploinsufficiency of the *C9ORF72* gene, resulting in a reduced level of C9orf72 protein expression. Second, a toxic gain of function is documented through repeat RNA foci that bind and sequester essential RNA-binding proteins (Conlon et al., 2016; Cooper-Knock et al., 2014; Lee et al., 2013), causing dysregulation of RNA metabolism (Cooper-Knock et al., 2015). Finally, five different forms of dipeptide repeat (DPR) proteins can be translated from the hexanucleotide repeat mRNA and form cytosolic aggregates (Freibaum and Taylor, 2017). In all, it seems that *C9ORF72* ALS (C9-ALS) results in a combination of loss and gain of function, although the exact contributions remain unknown.

While a large portion of ALS research has focused on motor neuron degeneration, recent observations support the idea that ALS pathology is not confined to motor neurons. In fact, several additional cell types have been shown to be involved in the ALS disease state, such as sensory neurons (Vaughan et al., 2018), mast cells and neutrophils (Trias et al., 2018), microglia, astrocytes and T cells (Rizzo et al., 2014). Furthermore, there has been increasing evidence that skeletal muscle is affected early in the ALS disease process, prior even to motor neuron cell death. Interestingly, motor neuron cell death occurs in a retrograde manner, beginning distally at the neuromuscular junction (NMJ) before spreading to the soma (Moloney et al., 2014; Krakora et al., 2012; Fischer et al., 2004). Therefore, understanding skeletal muscle pathology could help elucidate early disease processes occurring at the NMJ. Studies examining skeletal muscle in ALS mouse models have found changes at the presymptomatic stage, including fiber-type transitions, changes in the levels of myogenic regulatory factors, and abnormal mitochondrial morphology and function (Loeffler et al., 2016; Pansarasa et al., 2014). Early symptomatic muscle samples from human ALS patients also show mitochondrial abnormalities and changes in fiber types (Pansarasa et al., 2014). While protein aggregation is a major component to the neuropathology of ALS (Baloh, 2011; Neumann et al., 2006, 2007; Gao et al., 2018), it has only recently begun to be investigated in ALS skeletal muscle. For example, TDP-43, an RNA- and DNA-binding protein that is mutated in certain forms of familial ALS, is commonly found in cytosolic aggregates in ALS patient neurons regardless of genetic background (Gao et al., 2018). TDP-43 aggregation was recently discovered to be present in ALS patient muscle biopsies as well, including some with the *C9ORF72* mutation (Cykowski et al., 2018). So far, a mechanistic link has not been established between TDP-43 aggregation and the *C9ORF72* mutation.

Induced pluripotent stem cells (iPSCs) represent an opportunity to model early skeletal muscle pathology *in vitro*. Recent advances in iPSC technology allow for the creation of patient-derived stem cells, which have become a valuable resource for preparing different types of cells, including skeletal myocytes. Many variations of skeletal myocyte differentiation protocols have been developed (Chal and Pourquié, 2017; Kodaka et al., 2017; Jiwlawat et al., 2018), and some have been used on ALS patient iPSCs (Lenzi et al., 2016; Swartz et al., 2016). However, the mechanisms by which ALS mutations cause skeletal muscle pathology have yet to be characterized. In the current study, patient-derived C9-ALS iPSCs were differentiated into myogenic progenitors and skeletal myocytes using a transgene-free approach (Hosoyama et al., 2014; Jiwlawat et al., 2017) and then analyzed for signs of *C9ORF72*-linked pathology, including haploinsufficiency, RNA foci and DPR proteins. We did not find any loss of C9orf72 protein from haploinsufficiency, but we did find RNA foci and DPR protein aggregates. RNA sequencing found changes in the expression of genes related to mitochondrial function, results that were supported by an increased susceptibility to oxidative stress. The expression levels of other ALS-related genes, such as *TARDBP* and *SIGMAR1*, were changed in C9-ALS skeletal myocytes as well. Finally, aggregation of phosphorylated TDP-43 was found in C9-ALS skeletal myocytes. Together, these results show the feasibility of iPSC-derived skeletal myocytes for *in vitro* disease modeling of ALS and support the hypothesis that skeletal muscle experiences cell-autonomous pathology early in the ALS disease process.

## RESULTS

### C9-ALS iPSCs could be successfully differentiated into mature skeletal myocytes

**Fig. 1.**
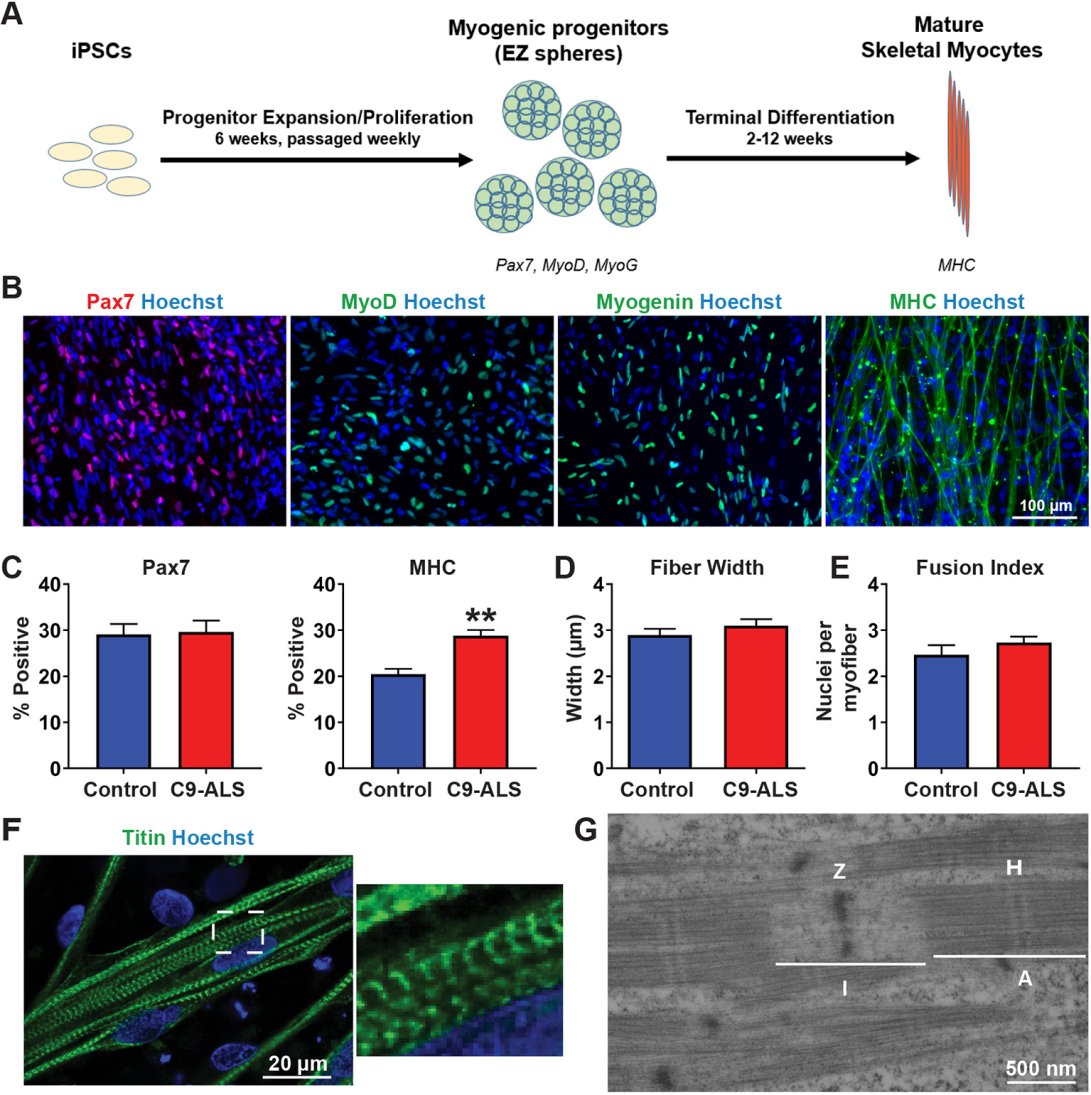
**C9-ALS-patient iPSCs are able to differentiate into mature skeletal myocytes.** (A) The differentiation process from iPSCs to myogenic progenitors, and then to mature skeletal myocytes using a transgene-free, sphere-based approach. (B) Representative images of muscle markers Pax7, MyoD, myogenin (MyoG) and myosin heavy chain (MHC) on day 14 of terminal differentiation. (C) Percentage of cells positive for Pax7 or MHC at the day-14 time point. (D) Average fiber width of skeletal myocytes at the day-14 time point. (E) The fusion index of day-14 skeletal myocytes. Error bars represent s.e.m. of values from at least three replicate experiments of at least two cell lines. **P<0.01, as determined by unpaired Student's *t-*test. (F,G) Sarcomere formation at the 6-week time point as shown by titin stain (F) and electron microscopy (G). A, A band (overlap of thick myosin filaments and thin actin filaments); H, H-zone (myosin filaments); I, I band (actin filaments); Z, Z line (defining border of a sarcomere unit).

We first confirmed whether C9-ALS iPSCs can form mature skeletal myocytes using our culture method for skeletal muscle differentiation. The iPSCs were differentiated using a transgene-free protocol as described previously in our recent publications (Hosoyama et al., 2014; Jiwlawat et al., 2017) (Fig. 1A). Patient-derived iPSCs were grown in suspension with a high concentration of epidermal growth factor (EGF) and basic fibroblast growth factor (FGF-2) to form myogenic progenitor cells as spherical aggregates. These myogenic progenitors were then dissociated and plated down for terminal differentiation. After 2 weeks, cells were fixed and stained for a myogenic progenitor marker (Pax7), a myoblast marker (MyoD), a committed myocyte marker (myogenin; MyoG) and a differentiated myocyte marker myosin heavy chain (MHC) (Buckingham and Mayeuf, 2012) (Fig. 1B). These data demonstrate that C9-ALS iPSCs can successfully differentiate into skeletal myocytes.

To compare the ability of each line to differentiate into mature multinucleated skeletal myocytes, we quantified the number of Pax7-positive and MHC-positive nuclei as well as the average fiber width and fusion index. Although there were variations in the data from individual cell lines for each characterization (Fig. S1), we did not see an overall significant difference in the number of Pax7-positive cells (Fig. 1C), the fiber width (Fig. 1D) or the fusion index (Fig. 1E) when comparing the average of control and C9-ALS lines. Interestingly, the C9-ALS lines, when grouped together, had a significantly higher amount of MHC-positive cells than control lines (Fig. 1C). Together, these results support that the *C9ORF72* mutation does not negatively impact myogenesis. Our previous work has shown that long-term culture promotes further maturation of myocytes differentiated using our protocol, including spontaneous contractions and organized sarcomere structure (Jiwlawat et al., 2017). Accordingly, spontaneous contractions could be observed starting around 4 weeks of differentiation, and contractions could be stimulated by adding caffeine to the culture media. In both cases, the control cells appeared to have more coordinated contractions (Movies 1 and 3), whereas the C9-ALS myocytes had individual fibers contracting randomly (Movies 2 and 4). After 6 weeks of terminal differentiation, myocytes show organized sarcomere structures as indicated from immunostaining for titin (Fig. 1F) as well as electron microscopy (Fig. 1G). In all, C9-ALS iPSC lines were able to terminally differentiate into skeletal myocytes with a maturation level similar to healthy controls. Further, our data indicate that the *C9ORF72* mutation does not hinder myogenesis but may affect contraction dynamics.

### C9-ALS skeletal myocytes do not experience decreased C9orf72 protein expression due to haploinsufficiency

**Fig. 2.**
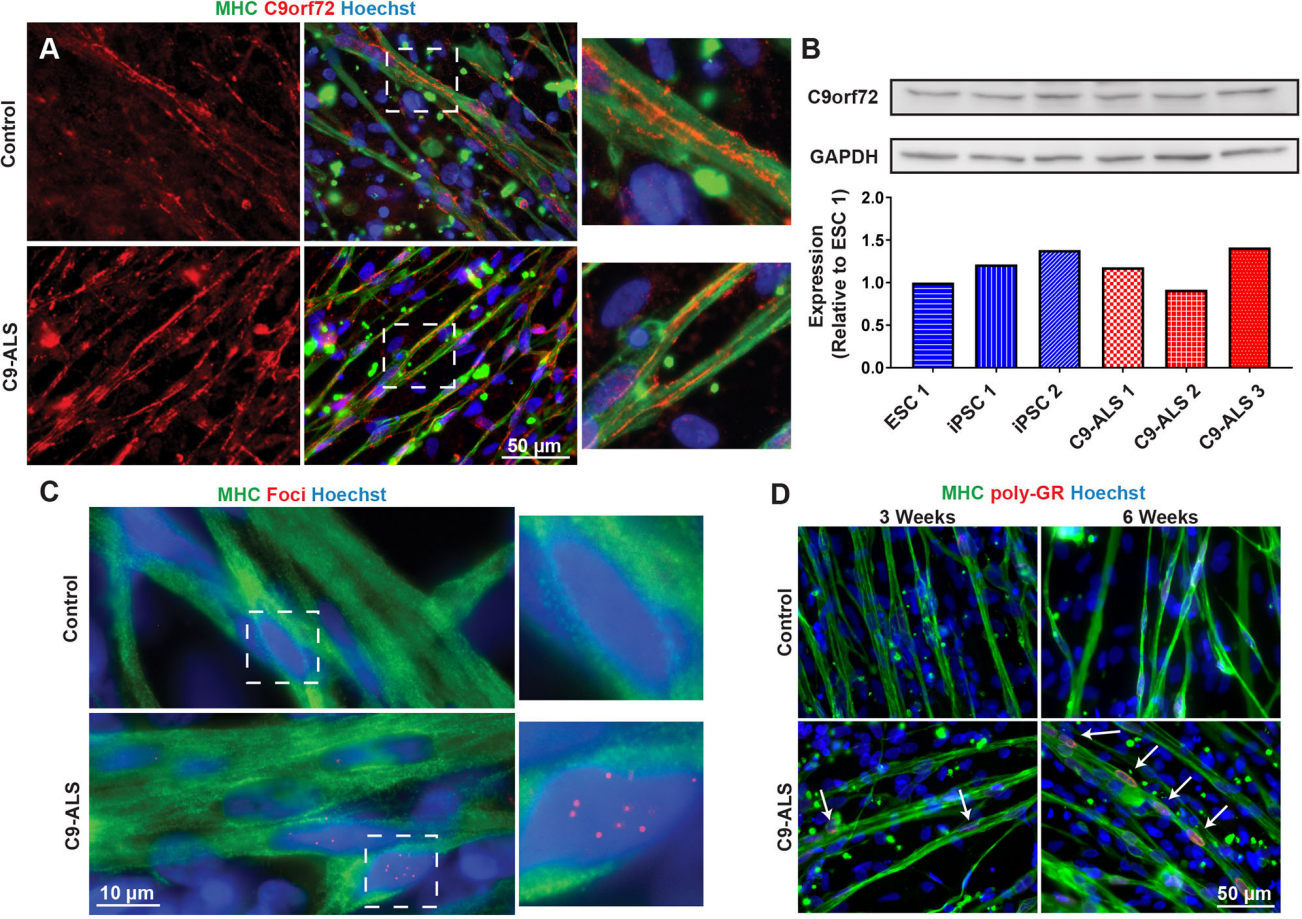
**C9-ALS skeletal myocytes do not have a loss of C9orf72 protein but do contain RNA foci and DPR protein aggregation.** (A) Representative images of immunostaining for C9orf72 protein expression in control and C9-ALS myocytes at the 6-week time point. (B) Western blot for C9orf72 protein expression and quantification confirms comparable expression between C9-ALS and control lines, indicating a lack of haploinsufficiency. Not significant through one-way ANOVA. (C) Fluorescence *in situ* hybridization (FISH) for GGGGCC-repeating RNA foci shows RNA foci in both the nuclei and cytoplasm of C9-ALS myocytes but not controls. (D) Staining for the DPR protein poly-GR shows primarily nuclear localization (arrows) that increases over time.

Next, we asked whether C9-ALS iPSC-derived skeletal myocytes exhibited the hallmark signs of cellular pathology associated with the *C9ORF72* mutation, including haploinsufficiency, RNA foci and DPR proteins (Moens et al., 2017). First, we determined whether C9-ALS myocytes had decreased C9orf72 protein expression as a result of possible haploinsufficiency caused by the repeat expansion. To test this, we performed immunocytochemistry and western blot for the C9orf72 protein. Immunocytochemistry showed similar expression levels of the C9orf72 protein in C9-ALS and control myocytes (Fig. 2A). This result was confirmed using three anti-C9orf72 antibody clones from different recourses in each cell line (Fig. S2). Western blot results also showed comparable C9orf72 protein expression between lines (Fig. 2B). Together, these results suggest that haploinsufficiency is not a major pathogenic mechanism in C9-ALS muscle, as C9orf72 protein seems to have similar expression in both healthy and C9-ALS myocytes. We did find an interesting localization of the C9orf72 protein on the periphery of the myocytes (Fig. 2A) that should be investigated further with regards to the function of the C9orf72 protein in skeletal muscle cells, which is currently not well defined.

### C9-ALS skeletal myocytes contain RNA foci and aggregates of the DPR protein poly-GR

Another possible effect of the *C9ORF72* repeat expansion on cellular pathology is the presence of repeat RNA foci. When the *C9ORF72* gene is transcribed to RNA, it adopts a secondary structure to form RNA foci, which have been shown in other cell types to bind and sequester RNA-binding proteins (Cooper-Knock et al., 2014; Conlon et al., 2016; Lee et al., 2013). In order to check whether these foci are present in skeletal myocytes, we used RNA fluorescence *in situ* hybridization (RNA FISH) to detect GGGGCC-repeating RNA foci. Foci were found in both the nuclei and cytoplasm of C9-ALS myocytes but not in controls (Fig. 2C). There were significantly more foci in the nuclei (2.95±0.82 foci per image; *n*=60 images) than the cytoplasm (1.27±0.20 foci per image; *n*=60 images, *P*<0.05). Interestingly, some nuclei contain several foci while another nucleus of the same myotube may contain none (Fig. 2C). For nuclei containing RNA foci (*n*=52 nuclei), the average number of foci per nucleus was 3.42, with a mode of 1 and maximum of 37.

The third hallmark of the *C9ORF72* repeat expansion is the aggregation of DPR proteins. The repeat expansion can undergo repeat-associated non-ATG (RAN) translation in the sense and antisense direction, allowing for five possible DPR species depending on where the translation begins (Freibaum and Taylor, 2017). In order to test whether skeletal myocytes contain DPR aggregates, we performed immunostaining for the DPR proteins poly-GR, poly-GP and poly-GA. Several antibodies against poly-GR, poly-GA and poly-GP were tested (Table S1). While all antibodies showed a speckled positive signal at the nuclei with occasional cytoplasmic positive signals (Fig. S3), only poly-GR was consistently stronger in C9-ALS lines than controls, and seemed to increase over time (Fig. 2D, Fig. S4).

### RNA sequencing reveals changes in genes that regulate mitochondrial function

**Fig. 3.**
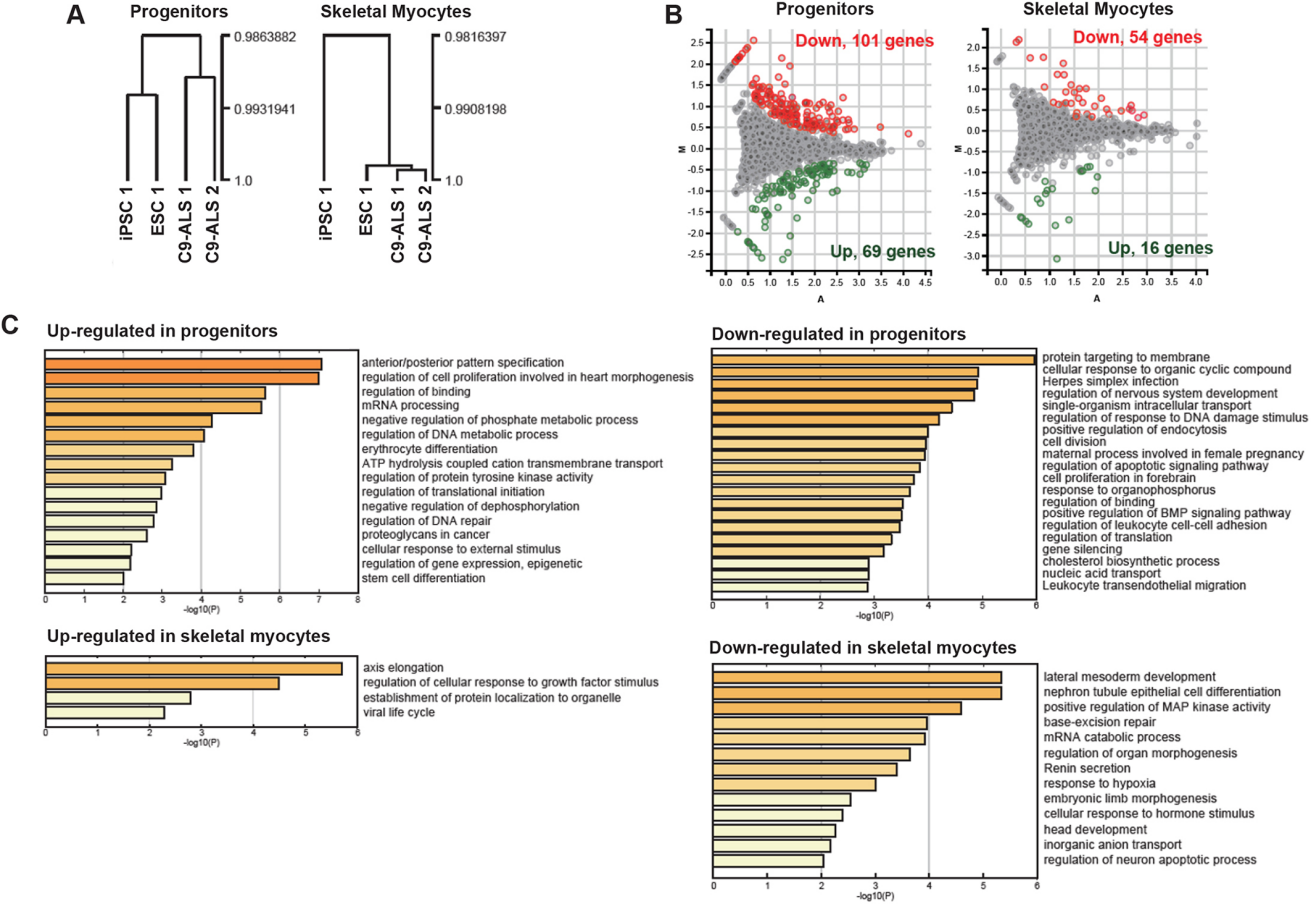
**Transcriptional characterization of myogenic progenitors and day-14 skeletal myocytes derived from C9-ALS iPSCs.** (A) Cluster analysis of gene expression of C9-ALS cells. The ordinates represent the Pearson's correlation coefficient value. (B) Differential gene expression between C9-ALS and control cells visualized by MA plot. Genes that were differentially expressed between groups are indicated in red and green (FDR ≤0.05, fold change ≥1.5). (C) Gene ontology and pathway analysis of differentially expressed genes in C9-ALS progenitors and myocytes.

To reveal the gene expression profile of C9-ALS muscle cells, we performed transcriptome analysis (RNA-Seq) of myogenic progenitors and day-14 differentiated myocytes derived from two lines of C9-ALS iPSCs (C9-ALS 1 and C9-ALS 2) and compared them to an embryonic-stem-cell and iPSC control (ESC 1 and iPSC 1). Clustering analysis revealed that C9-ALS cells showed very similar patterns of gene expression compared to control cells in both stages (Pearson correlation coefficient >0.98) (Fig. 3A). Although the overall pattern of gene expression was similar between C9-ALS and control lines, a specific set of genes was differentially expressed. Detailed analysis revealed that 69 and 101 genes were up- and down-regulated, respectively, in C9-ALS progenitor cells. In addition, 16 and 54 genes were up- and down-regulated, respectively, in C9-ALS differentiated myocytes (Fig. 3B). Next, we identified specific roles of the genes differentially expressed between C9-ALS and control cells by gene ontology enrichment analysis. We found that these genes were involved in various biological functions. For instance, there were genes responsible for anterior/posterior pattern specification and protein targeting to membrane in the gene list of myogenic progenitor samples. In C9-ALS differentiated myocytes, some genes related to axis elongation were upregulated. On the other hand, the expression of genes for lateral mesoderm development was decreased (Fig. 3C).

**Fig. 4.**
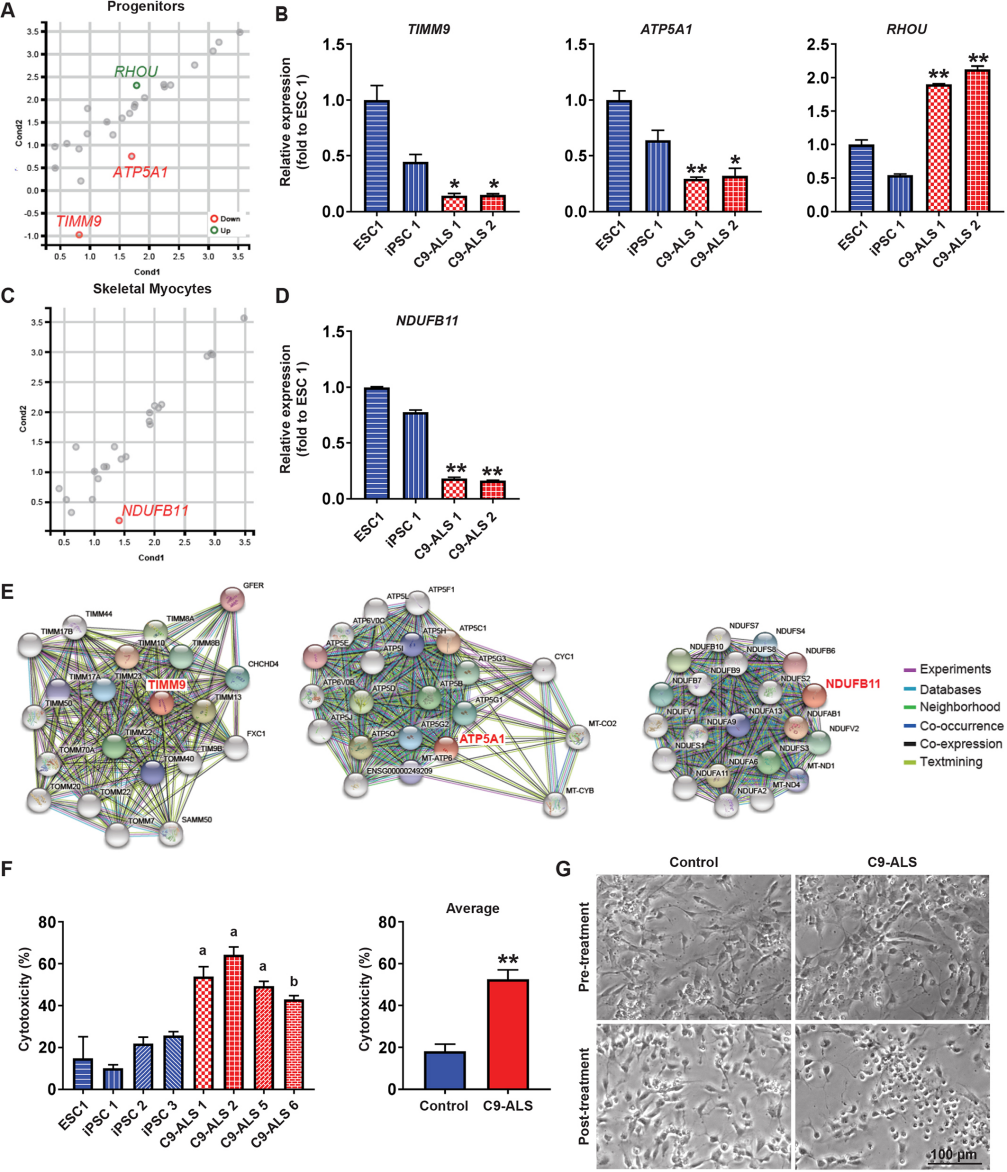
**C9-ALS progenitors and skeletal myocytes show signs of mitochondrial dysfunction and susceptibility to oxidative stress.** (A,C) Scatterplot of gene expression comparing C9-ALS and control cells. Only genes categorized as mitochondrial organization by gene ontology were visualized. Genes that were differentially expressed between groups are indicated in red and green (FDR ≤0.05, fold change ≥1.5). (B,D) RT-qPCR confirmed differential expression of genes related to mitochondrial function. Error bars represent s.e.m. from three technical replicates. *P*<0.05, * significantly different from one control, ** significantly different from both controls as determined by one-way ANOVA followed by Tukey's post-hoc multiple-comparisons test. (E) Association networks of up- or down-regulated genes in C9-ALS cells using STRING software. Lines indicate protein-protein associations that are meant to be specific and meaningful. (F) Percent toxicity as measured by LDH release from myogenic progenitors treated with hydrogen peroxide. Error bars represent s.e.m. from three technical replicates. a: significant compared to all four controls; b: significant compared to all controls except iPSC 3. Considered significant if *P*<0.05 as determined by one-way ANOVA followed by Tukey's post-hoc multiple-comparisons test. Average: **P<0.01, unpaired Student's *t*-test. (G) Live imaging of myogenic progenitors immediately following the 30-min incubation with hydrogen peroxide, showing more severe morphological changes in C9-ALS cells.

Particularly in these gene profiles, specific genes categorized as mitochondrial organization showed differential expression in C9-ALS cells (Fig. 4A,C). Expression of *RHOU* (Ras homolog family member U) was significantly increased, whereas *TIMM9* (translocase of inner mitochondrial membrane 9) and *ATP5A1* (ATP synthase F1 subunit alpha) were significantly decreased in C9-ALS myogenic progenitors (Fig. 4A), and *NDUFB11* (NADH:ubiquinone oxidoreductase subunit B11) was significantly decreased in C9-ALS myocytes (Fig. 4C). We used reverse-transcription qPCR (RT-qPCR) to confirm the expression levels for each of these genes (Fig. 4B,D). Next, we analyzed the functional network of the three downregulated genes (*TIMM9*, *ATP5A1* and *NDUFB11*) in C9-ALS cells using STRING software (Fig. 4E). TIMM proteins are located in the inner mitochondria membrane and play an essential role in the import of cytosolic proteins (Bauer et al., 2000). Since TIMM9 interacts with other TIMM family members, such as TIMM22 and TIMM23, the decrease of TIMM9 expression in C9-ALS myocytes may cause the inactivation of protein import by the TIMM complex. On the other hand, ATP5A1 and NDUFB11 commonly have functional interactions with mitochondrial metabolic factors such as MT-CO2 (mitochondrially encoded cytochrome c oxidase II), MT-CYB (mitochondrially encoded cytochrome b), MT-ND1 (mitochondrially encoded NADH dehydrogenase 1) and MT-ND4 (mitochondrially encoded NADH dehydrogenase 4), suggesting a possible involvement of these molecules in dysregulation of mitochondrial function in C9-ALS cells.

### C9-ALS iPSC-derived myogenic progenitors display an increased susceptibility to oxidative stress

RNA-Seq results indicate that differential expression of the above-mentioned genes may be implicated in C9-ALS-linked cellular pathology such as mitochondrial dysfunction. Mitochondria are a major producer of reactive oxygen species (ROS) and are involved in the regulation of oxidative stress, which is a common factor in many neurodegenerative diseases (Keating, 2008; Ott et al., 2007). To test whether the *C9ORF72* mutation leads to oxidative stress in skeletal myocytes, C9-ALS myogenic progenitors were plated on coverslips and then treated with hydrogen peroxide (H_2_O_2_) for 30 min (Fig. 4F,G). The cells were returned to the terminal differentiation medium and imaged for morphological changes. The myogenic progenitors of both the control lines and C9-ALS lines retracted their processes and became more balled up. However, this phenotype seemed more severe in the C9-ALS cells (Fig. 4G). After 24 h, the conditioned medium was collected and used to quantify the amount of cytotoxicity by measuring the amount of lactate dehydrogenase (LDH) release (Fig. 4F). The C9-ALS myogenic progenitors had a significantly higher amount of LDH release than controls, indicating an inherent susceptibility to oxidative stress. The susceptibility to oxidative stress of the C9-ALS lines was also compared to additional ALS patient iPSC lines, including *FUS*, *SOD1*-N193K, *SOD1*-A4V and a sporadic line (Fig. S5). Together with the RNA-Seq results, the increased cytotoxicity in response to exogenous oxidative stress may be caused by the inherent mitochondrial abnormalities resulting in a reduced ability to survive oxidative stress.

### C9-ALS myocytes have changes in the expression level of ALS-causing genes and aggregation of TDP-43

**Fig. 5.**
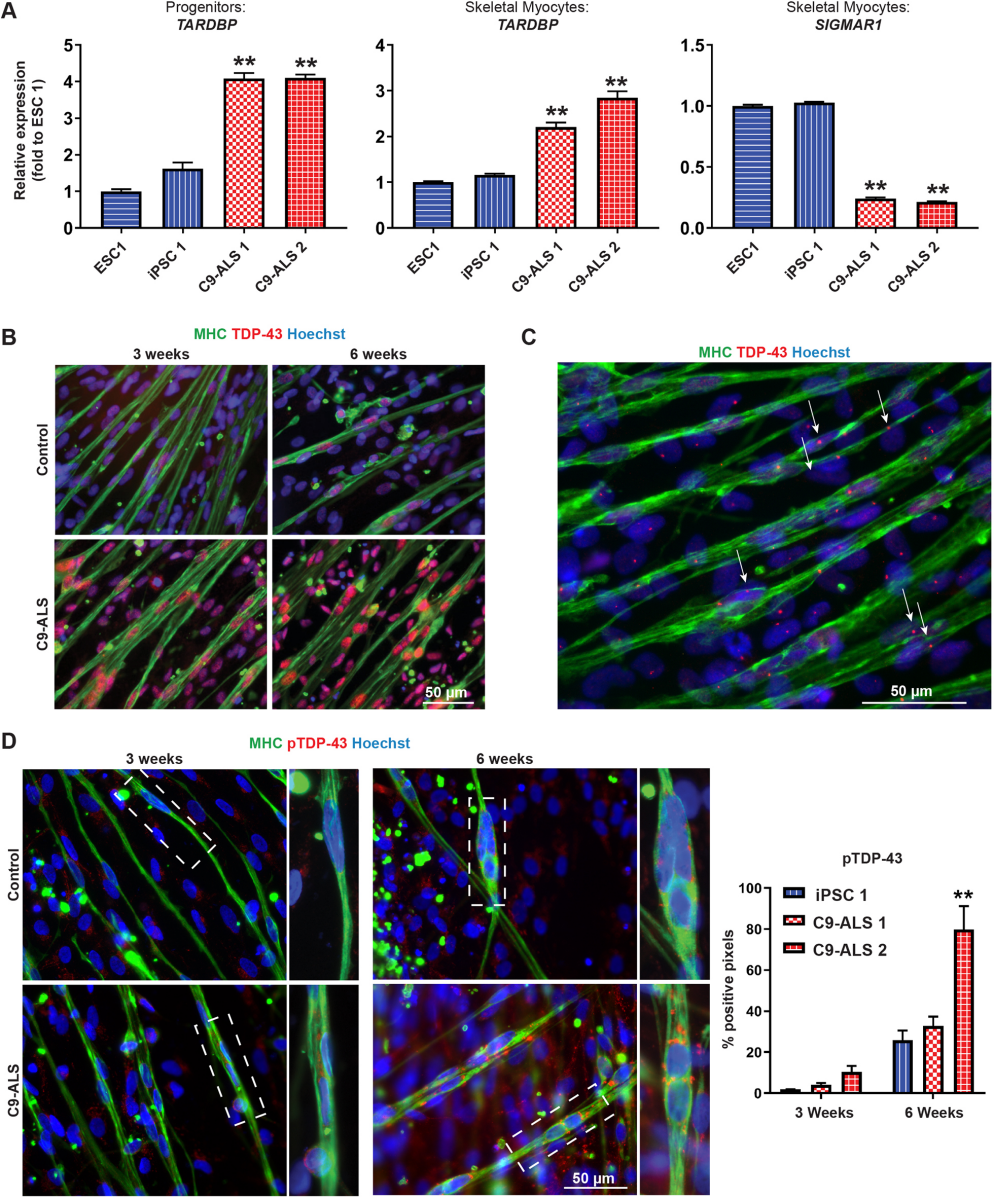
**C9-ALS skeletal myocytes have changes in expression of familial ALS genes and TDP-43 aggregation.** (A) RT-qPCR confirms changes in the expression level of ALS-causing genes *SIGMAR1* and *TARDBP*. Error bars represent s.e.m. from three technical replicates. **P<0.05, significantly different from both controls as determined by one-way ANOVA followed by Tukey's post-hoc multiple-comparisons test. (B) TDP-43 immunostaining at 3 weeks and 6 weeks post-terminal differentiation shows increased expression in C9-ALS myocytes. (C) Intranuclear aggregates of TDP-43 (arrows) in one of the C9-ALS lines. (D) Representative images of phosphorylated TDP-43 aggregates, which appear to increase over time, as supported by pixel analysis from a representative trial. Error bars represent s.e.m. from six analyzed images. **P<0.01 as determined by one-way ANOVA followed by Tukey's post-hoc multiple-comparisons test.

An additional interesting finding from RNA-Seq that was confirmed using RT-qPCR was the differential gene expression of ALS-related genes, such as *SIGMAR1* and *TARDBP* (Fig. 5A). *SIGMAR1* encodes sigma-1 receptor, an endoplasmic reticulum (ER) chaperone protein localized to the mitochondria-associated ER membrane (Penke et al., 2018). Mutations in *SIGMAR1* have been linked to early-onset ALS (Al-Saif et al., 2011). RT-qPCR confirmed RNA-Seq results that *SIGMAR1* expression is downregulated in C9-ALS skeletal myocytes. Conversely, *TARDBP* expression was upregulated. *TARDBP* encodes TDP-43, a DNA- and RNA-binding protein that regulates mRNA metabolism and processing. In ALS patients, TDP-43 moves from its primary location in the nucleus to the cytoplasm, where it undergoes modifications such as truncation or phosphorylation and forms cytosolic aggregates (Gao et al., 2018). This has been well characterized in neurons, but only recently detected in ALS-patient skeletal muscle (Cykowski et al., 2018). Since TDP-43 mislocalization and aggregation is a common feature of ALS regardless of genetic background, we wanted to see whether C9-ALS skeletal myocytes show signs of TDP-43 pathology. Immunocytochemistry for full-length TDP-43 found a stronger signal of nuclear TDP-43 in C9-ALS lines compared to controls at both 3 weeks and 6 weeks of terminal differentiation (Fig. 5B, Fig. S6), which supports the changes in gene expression levels detected using RNA-Seq and RT-qPCR. Additionally, one line (C9-ALS 2) consistently showed nuclear aggregates of TDP-43 (Fig. 5C), while other C9-ALS lines occasionally but not always exhibited this phenomenon (data not shown). We also detected an increase in phosphorylated TDP-43 expression in the C9-ALS cells. Aggregates of phosphorylated TDP-43 were present in the cytoplasm, particularly in the area adjacent to the nuclei (Fig. 5D). This aggregation was time dependent, with some noticeable aggregation at the 3-week time point but much more prominent aggregates at the 6-week time point. These results indicate that C9-ALS skeletal myocytes are affected by TDP-43-related pathology.

## DISCUSSION

Evidence suggests that ALS skeletal muscle experiences pathological changes early in the disease process, prior to motor neuron degeneration and symptom onset (Pansarasa et al., 2014; Loeffler et al., 2016; Moloney et al., 2014; Fischer et al., 2004). Therefore, studying the pathological mechanisms that are occurring in skeletal muscle could help to discover early detection methods or new therapeutic options. ALS-patient iPSCs provide a model of patient-specific skeletal muscle abnormality. So far, there have been very few studies that have utilized iPSCs for the study of ALS skeletal muscle. One used MyoD induction to differentiate skeletal myocytes from iPSCs of patients with *FUS* and *TARDBP* mutations, and found some physiological deficits (Lenzi et al., 2016). Another used a small-molecule-based protocol to differentiate *C9ORF72* patient iPSCs into skeletal myocytes and found RNA foci but no signs of TDP-43 pathology or any additional defects (Swartz et al., 2016). Our study builds on this basis of C9-ALS skeletal myocyte pathology by showing signs of DPR protein expression, changes in genes involved in mitochondrial function, a susceptibility to oxidative stress and evidence of TDP-43 proteinopathy. It is difficult to pinpoint why our iPSC-derived skeletal myocytes showed more pathological abnormalities than the previous study. It could be due to many variables, including the differentiation protocol, time points or patient backgrounds.

In the current study, we first confirmed that C9-ALS iPSCs can differentiate into myogenic progenitors and mature functional skeletal myocytes using our transgene-free differentiation protocol (Hosoyama et al., 2014). We found that these cells differentiated and matured similarly to control lines, which suggests that the *C9ORF72* mutation does not negatively affect skeletal muscle differentiation or development. However, we did find that the C9-ALS myocytes contracted in a less coordinated manner than control cells.

These differentiated myocytes were used to study the three hallmarks of C9-ALS: haploinsufficiency, repeat RNA foci and DPR proteins. In the literature there has been conflicting studies regarding whether haploinsufficiency occurs in ALS patients. Interestingly, animal models containing a knockout of the C9orf72 protein do not experience motor dysfunction (Burberry et al., 2016; O'Rourke et al., 2016; Sudria-Lopez et al., 2016), suggesting that a loss of function due to haploinsufficiency would not be a main pathogenic mechanism in C9-ALS patients. However, a recent study found that *C9ORF72* haploinsufficiency causes decreased endosomal trafficking and lysosomal function, which leads to excitotoxicity and a build-up of toxic DPR proteins in an iPSC-derived motor neuron cell model (Shi et al., 2018).

It is unknown whether C9-ALS skeletal muscle cells experience haploinsufficiency. One case study did find a loss of C9orf72 protein expression in a patient autopsy muscle sample (Türk et al., 2014). The previous study using C9-ALS iPSC-derived skeletal myocytes found no change in mRNA expression of the three C9orf72 protein variants compared to controls (Swartz et al., 2016). Corresponding to their results, we did not find any obvious evidence of haploinsufficiency in our patient-iPSC-derived skeletal myocytes, as expression levels of the C9orf72 protein were comparable to control lines when measured using immunocytochemistry and western blot. The biological function of the C9orf72 protein in skeletal muscle is currently unknown. The C9orf72 protein has been structurally linked to DENN proteins, which regulate membrane vesicle trafficking (Levine et al., 2013; Zhang et al., 2012), and several papers support a role of the C9orf72 protein in vesicle trafficking and lysosomal function (Amick et al., 2016; Farg et al., 2014; Sellier et al., 2016; Webster et al., 2016a,b; Yang et al., 2016; Shi et al., 2018). Based on those studies and the peripheral localization of the C9orf72 protein in our patient-iPSC-derived skeletal myocytes, future studies on the function of the C9orf72 protein in skeletal muscle should investigate deficits in vesicle trafficking and whether the protein interacts with receptors on the surface of skeletal muscle.

Although haploinsufficiency was not identified in our patient-iPSC-derived myocytes, we were able to detect repeat RNA foci in both the cytoplasm and nuclei. It remains to be seen whether skeletal muscle experiences downstream pathology as a result of the RNA foci, such as sequestration of RNA-binding proteins and splicing irregularities, as has been found in motor neuron models (Lee et al., 2013; Cooper-Knock et al., 2014, 2015; Conlon et al., 2016). We were also able to detect the DPR protein poly-GR in the skeletal myocytes. To our knowledge, no other studies have detected DPR proteins in human skeletal muscle. However, a study of *Caenorhabditis elegans* overexpressing (PR)_50_ or (GR)_50_ found nuclear localization of these proteins in the skeletal muscle (Rudich et al., 2017), which is in agreement with the localization that we found in the iPSC-derived skeletal myocytes. Our findings of RNA foci and DPR protein aggregates but no haploinsufficiency suggest that skeletal myocytes may be affected by the *C9ORF72* mutation through a toxic gain of function (protein aggregation and RNA toxicity) rather than a loss of function (haploinsufficiency). It is possible that these toxic gain-of-function mechanisms may be interlinked. For example, a recent study using a mouse model of poly-PR toxicity found that poly-PR interferes with heterochromatin structural organization, resulting in RNA toxicity due to increased repetitive elements and accumulation of double-stranded RNA (Zhang et al., 2019). Downstream effects of RNA foci and DPR aggregation are yet to be determined in the context of skeletal muscle.

We next used RNA sequencing to determine whether C9-ALS myocytes have any changes in gene expression that may also be affecting the disease state. RNA-Seq results found changes in the expression levels of several genes related to mitochondrial function, including *RHOU*, *TIMM9* and *ATP5A1*. These gene-expression changes were further confirmed using RT-qPCR. Together, these results suggest that C9-ALS skeletal myocytes may have defects in mitochondrial function. This is supported by a recent study using an inducible mouse model expressing poly(GR)_80_ that found that poly-GR binds to the mitochondrial subunit ATP5A1 and enhances its degradation, resulting in mitochondrial dysfunction (Choi et al., 2019). Mitochondrial dysfunction can lead to over-production of ROS, causing oxidative stress, a common feature of neurodegenerative diseases (Briston and Hicks, 2018). Skeletal muscle has been found to experience oxidative stress prior to motor impairment in a mouse model of ALS (Halter et al., 2010). Our results show that C9-ALS lines have an increased susceptibility to exogenous oxidative stress at the progenitor stage, possibly implying an inherent susceptibility to oxidative stress in the skeletal muscle of ALS patients. This is consistent with a study that found decreased mitochondrial function and increased levels of ROS in iPSC-derived motor neurons (Lopez-Gonzalez et al., 2016). Together, these findings suggest that oxidative stress could be a systemic occurrence in C9-ALS patients.

Interestingly, RNA-Seq results also found that expression levels were changed for two familial ALS-related genes, *TARDBP* and *SIGMAR1*. To further expand on our results from RNA-Seq and RT-qPCR that *TARDBP* expression levels were increased, we stained C9-ALS skeletal myocytes for full-length TDP-43 and phosphorylated TDP-43. We found an increase in full-length TDP-43 in the nuclei of C9-ALS myocytes, as well as aggregates of phosphorylated-TDP-43 expression in the cytoplasm of C9-ALS cells. We also occasionally noted intranuclear aggregates of TDP-43 in the C9-ALS lines. So far, very little is known about the role of TDP-43 in skeletal muscle physiology or pathology. TDP-43 has been shown to have a role in synaptic function (Ling, 2018) and to interact with cytoskeletal components as well as the nuclear pore complex and nucleocytoplasmic transport proteins in neurons (Oberstadt et al., 2018; Chou et al., 2018). Overexpression or knockout of TDP-43 in mice results in changes in fat deposition and glucose homeostasis (Stallings et al., 2013). Interestingly, TDP-43 has been found to aggregate in the skeletal muscle of patients with non-ALS myopathies (Olivé et al., 2009; Salajegheh et al., 2009). More recently, TDP-43 aggregation has also been identified in the skeletal muscle of ALS patients and animal models (Wang et al., 2017; Cykowski et al., 2018). However, the link between TDP-43 pathology and the *C9ORF72* mutation has not yet been characterized.

The *C9ORF72* repeat expansion can result in ALS, frontotemporal dementia (FTD), or a combination of psychiatric and motor symptoms (Rohrer et al., 2015). Therefore, future studies similar to this one could investigate differences between *C9ORF72* iPSC-derived skeletal myocytes from patients with FTD, ALS, or with symptoms of both. Then we can possibly determine what causes the skeletal muscle to be specifically affected in one patient but not another. For the purpose of our current study, we focused on C9-ALS-patient iPSC-derived skeletal myocytes for *in vitro* disease modeling of early skeletal muscle pathology. C9-ALS lines differentiated into skeletal myocytes just as well as healthy control cell lines and showed signs of C9-ALS pathology as early as 3 weeks post-differentiation. This raises an interesting point that skeletal muscle could be experiencing pathology much earlier in the disease process than was previously assumed. While there is a need to confirm these signs of pathology in ALS-patient muscle tissue, the present study supports that skeletal muscle experiences cell-autonomous pathology in ALS patients. Cross-talk between skeletal muscle and an adjacent motor neuron is a critical component of NMJ formation during development, and a continued part of NMJ maintenance (Cappello and Francolini, 2017). By understanding the pathological processes that are occurring in skeletal muscle at the presymptomatic stage, there is hope to better understand how skeletal muscle may contribute to NMJ degeneration and subsequent disease processes.

## MATERIALS AND METHODS

### Human pluripotent stem cells

Patient-derived iPSC lines with *C9ORF72* mutations were obtained from the Target ALS stem cell core at RUCDR Infinite Biologics (Target ALS IDs TALS9-9.3 and TALS9-9.5; they are referred as ‘C9-ALS 1’ and ‘C9-ALS 2’ in this study, respectively) (Piscataway Township, NJ, USA) and Cedars-Sinai iPSC stem cell core (four iPSC lines named 28i, 29i, 30i and 52i; referred to here as ‘C9-ALS 3, 4, 5 and 6’) (Los Angeles, CA, USA). A human embryonic stem cell line WA09 (‘ESC 1’) and human iPSC line IMR90 (‘iPSC 1’) were obtained from WiCell (Madison, WI, USA), and iPSC line TD-A-47 (‘iPSC 2’) was obtained from Cellular Dynamics International (Madison, WI, USA). Stem cell lines from Cedars-Sinai were cultured on a mouse embryonic fibroblast feeder layer (Thomson et al., 1998), and lines from WiCell and Target ALS were cultured using a feeder-free protocol (Ludwig et al., 2006a,b).

### Differentiation of human iPSCs to myogenic progenitors and mature skeletal myocytes

Human iPSCs were differentiated into myogenic progenitors and mature skeletal myocytes as previously described (Hosoyama et al., 2014; Jiwlawat et al., 2017). Briefly, iPSC colonies were dissociated using 2 mg/ml dispase (Life Technologies, Carlsbad, CA, USA) or 0.1% collagenase (Life Technologies). The lifted cells were rinsed once in Stemline medium (S-3194, Sigma-Aldrich, St Louis, MO, USA), then resuspended in an expansion medium consisting of a Stemline medium with 100 ng/ml human EGF (Millipore, Billerica, MA, USA), 100 ng/ml recombinant human FGF-2 (WiCell), 5 µg/ml heparin sulfate (Sigma-Aldrich) and 1% w/v penicillin/streptomycin/amphotericin B (PSA; Thermo Fisher Scientific, Waltham, MA, USA). Cells were grown in flasks coated with poly(2-hydroxyethyl methacrylate) (polyHEMA, Sigma-Aldrich) and cultured as free-floating spherical aggregates termed EZ spheres (Ebert et al., 2013; Hosoyama et al., 2014). These spheres were passaged by mechanical chopping using a McIlwain tissue chopper (Mickle Laboratory Engineering, Surrey, UK) once a week for 6-12 weeks.

For terminal differentiation into skeletal myocytes, spheres were dissociated using trypsin (TrypLE, Life Technologies) and plated onto coverslips pre-coated with poly-L-lysine (0.1 mg/ml, Sigma-Aldrich) and laminin (5 µg/ml, Sigma-Aldrich) at a density of 200,000 cells per coverslip. Cells were differentiated into myocytes in a skeletal muscle differentiation medium consisting of DMEM/GlutaMAX (10566-016, Life Technologies), 2% B-27 serum-free supplement (Life Technologies) and 1% PSA. Myocytes were cultured up to 12 weeks post-differentiation.

### Immunocytochemistry and image quantification

Cells were fixed with 4% paraformaldehyde (PFA; Electron Microscopy Sciences, Hatfield, PA, USA) in phosphate buffered saline (PBS) for Pax7, MyoD and myogenin stains, and ice-cold methanol for all others. Cells were permeabilized and blocked with 0.1% Triton X-100 (Sigma-Aldrich) and 5% normal donkey serum (NDS; Jackson ImmunoResearch, West Grove, PA, USA) in PBS for 20 min at room temperature. Primary and secondary antibodies were added at the dilutions as described in Table S1. Cell nuclei were labeled using Hoechst 33258 (0.5 µg/ml in PBS, Sigma-Aldrich). Coverslips were mounted on slides using a mounting medium (Fluoromount-G; SouthernBiotech, Birmingham, AL, USA). Stains were imaged using a Nikon Eclipse 80i fluorescent microscope (Nikon, Tokyo, Japan) with a DS-QilMC CCD camera (Nikon) or Leica TCS SP8 confocal microscope (Leica, St Galen, Switzerland).

For each plating of differentiated myocytes, cells were stained for Pax7 and MHC on day 14 of terminal differentiation. Each coverslip was imaged at six randomly selected fields of view at 20× magnification. From each image, the total number of nuclei and the number of nuclei positive for Pax7 (Pax7^+^) or within MHC-positive (MHC^+^) myocytes were counted. Pax7 expression levels were calculated as the average percentage of nuclei expressing Pax7, and MHC expression levels were calculated as the average percentage of nuclei within MHC^+^ myocytes for each field of view. The fiber width of at least three myocytes per image were also measured, and the fusion index was calculated as the number of nuclei contained within MHC^+^ myocytes divided by the total number of myocytes. The values graphed are the average of at least three separate trials per line, with six fields of view per trial. For the quantification of poly-GR, TDP-43 and pTDP-43 expression, each set of six images were analyzed for the percentage of positive pixels using the ‘Color Pixel Counter’ plugin on NIH ImageJ software.

### Western blot

Cells were lysed in radioimmunoprecipitation assay buffer (RIPA buffer; EMD Millipore, Burlington, MA, USA) with a protease inhibitor cocktail (Thermo Fisher Scientific) and 5 mM ethylenediaminetetraacetic acid (EDTA; Thermo Fisher Scientific). Protein concentrations were determined using the DC Protein Assay kit (Bio-Rad, Hercules, CA, USA). Proteins (20 μg per lane) were run on a polyacrylamide gel and transferred onto a PVDF membrane (EMD Millipore). The membrane was immunoblotted with anti-C9orf72 antibody (22637-1-AP, 1:500; ProteinTech Group, Inc., Rosemont, IL, USA) followed by secondary antibody conjugated with horseradish peroxidase (anti-rabbit IgG HRP; Promega, Madison, WI, USA). Enhanced chemiluminescence substrate (Pierce Biotechnology, Waltham, MA, USA) was used to detect HRP on the immunoblot for chemiluminescence imaging. Densitometry values of protein bands were analyzed by NIH ImageJ software.

### Skeletal muscle video capture

Skeletal muscle contractions were recorded using a Nikon Eclipse TS 100 inverted microscope and QImaging camera with Q Capture Pro Software (QImaging, Surrey, BC, Canada). Videos were recorded as .avi files at 10× magnification, with 400 frames at 20 frames per second. Contractions were either spontaneous or stimulated with 10 mM caffeine (Sigma-Aldrich).

### RNA fluorescence *in situ* hybridization (RNA FISH)

RNA FISH was conducted using a Cy3-tagged (GGGGCC)_4_ probe (Integrated DNA Technologies, Coralville, Iowa, USA) according to previously established protocols (Almeida et al., 2013; Liu et al., 2016). Cells were fixed with 4% PFA for 10 min and rinsed with PBS. Pre-chilled 70% ethanol was added to the cells and they were stored overnight at 4°C. Cells were rehydrated in 40% formamide (Sigma-Aldrich) and 2× saline-sodium citrate buffer (SSC; Promega) for 10 min at room temperature. Cells were then prehybridized for 15 min at 37°C in a humidified chamber in 40% formamide, 2× SSC, 10% dextran sulfate (EMD Millipore), 1 mg/ml yeast tRNA (Life Technologies) and 1 mg/ml salmon sperm DNA (Life Technologies). During this time the probe was denatured for 10 min at 100°C then put on ice for 10 min before being added to cold hybridization buffer and added to cells. The cells were hybridized for 2 h at 55°C in a humidified chamber then washed three times in 40% formamide and 1× SSC for 15 min at 55°C in a humidified chamber. Then cells were washed two times for 15 min at room temperature in 1× SSC. After being rinsed with PBS once, cells were then stained for MHC, as described above. Slides were imaged at 100× magnification and for each image the number of RNA foci present in myocyte nuclei and cytoplasm was counted and compared. The average number of foci within each nucleus was also calculated.

### Electron microscopy

Cells were fixed with 2% PFA and 2.5% glutaraldehyde (Electron Microscopy Sciences, Inc., Hatfield, PA, USA) in 0.1 M cacodylate buffer (Electron Microscopy Sciences, Inc.) overnight, followed by 1% osmium tetroxide for 1 h. Samples were then dehydrated with an ethanol gradient, embedded in Durcapan (Sigma-Aldrich), sectioned at 60 nm thickness, and stained with lead citrate and uranium acetate. Samples were imaged using a Philips CM120 transmission electron microscope (Eimdhoven, The Netherlands).

### Cell susceptibility assay against oxidative stress

Susceptibility to exogenous oxidative stress was measured as described previously (Li et al., 2012). Briefly, myogenic progenitors were plated down onto poly-L-lysine- and laminin-coated coverslips, and cultured in terminal differentiation medium in 24-well plates. After 24 h, the medium was removed and replaced by 100 μM H_2_O_2_ in PBS for 30 min then rinsed with PBS and replaced with fresh terminal differentiation media. Cells were imaged at this point in order to observe any immediate effects on cellular morphology. After 24 h, the conditioned media was collected and LDH release was measured using the CytoTox 96 Non-Radioactive Cytotoxicity Assay (Promega) and ChroMate spectrophotometer (Awareness Technology Inc., Palm City, FL, USA) with wavelength set to 492 nm.

### RNA sequencing

Total RNA was isolated from both myogenic progenitors and day-14 terminally differentiated skeletal myocytes using the Direct-zol RNA Kit (Zymo Research, Irvine, CA, USA) according to the manufacturer's instructions. RNA-Seq libraries were constructed from 500 ng of total RNA using KAPA RNA HyperPrep Kits with RiboErase (KAPA Biosystems, Wilmington, MA, USA) according to the manufacturer's instructions. Completed libraries were quantified using D1000 ScreenTape system (Agilent Technologies, Santa Clara, CA, USA) and were sequenced using Hiseq 4000 (Illumina, San Diego, CA, USA).

After pre-filtering the raw data by removing sequence adapters and low-quality reads, paired-end 100-bp sequences were aligned to assemble the human genome (hg38) by HISAT2 software in a Galaxy browser (https://usegalaxy.org/). Transcripts assembly, abundance and evaluation of differential expression were accomplished using the Cufflinks and DEseq software in a DEBrowser. Genes exhibiting a fold change ≥±1.5 and FDR <0.05 were considered differentially expressed in the cells derived from C9-ALS patients compared to two control cells (ESC 1 and iPSC 1). Gene ontology and protein-protein interactions were analyzed by Metascape and STRING software, respectively.

### Quantitative reverse transcription polymerase chain reaction (RT-qPCR)

Total RNA was isolated from cells using Direct-zol RNA Kit (Zymo Research) according to the manufacturer's instructions. First-strand cDNA was synthesized from 1 μg of total RNA using ReverTra Ace qPCR RT Master Mix with gDNA Remover (TOYOBO, Osaka, Japan).

RT-qPCR was performed with FastStart Essential DNA Green Master (Roche, Basel, Switzerland) using Light Cycler 96 (Roche). Primer sequences can be found in Table S2. PCR was performed with the following thermocycling conditions: denaturation at 95°C for 10 min and 45 cycles of denaturation at 95°C for 10 s, annealing at 58°C for 10 s and elongation at 72°C for 10 s. Data were normalized to the expression of *ACTB*.

### Statistical analysis

GraphPad Prism software (La Jolla, CA, USA) was used to perform statistical analyses. Graphs were presented as means±s.e.m. from at least three trials. One-way ANOVA was used to compare data points across cell lines. Differences were considered significant when *P*<0.05. A statistically significant one-way ANOVA was followed up with Tukey's post-hoc test for multiple comparisons. When control and C9-ALS lines were grouped as an average, unpaired Student's *t*-test was used to test for significance.

## Supplementary Material

Supplementary information
