# Supplementary material for: C9ORF72-related cellular pathology in skeletal myocytes derived from ALS-patient induced pluripotent stem cells
Source: Dis Model Mech. 2019 Aug 16;12(8):dmm039552. doi: 10.1242/dmm.039552 (PMC6737948; doi:10.1242/dmm.039552)
Supplement: Supplementary information [file dmm-12-039552-s1.pdf]

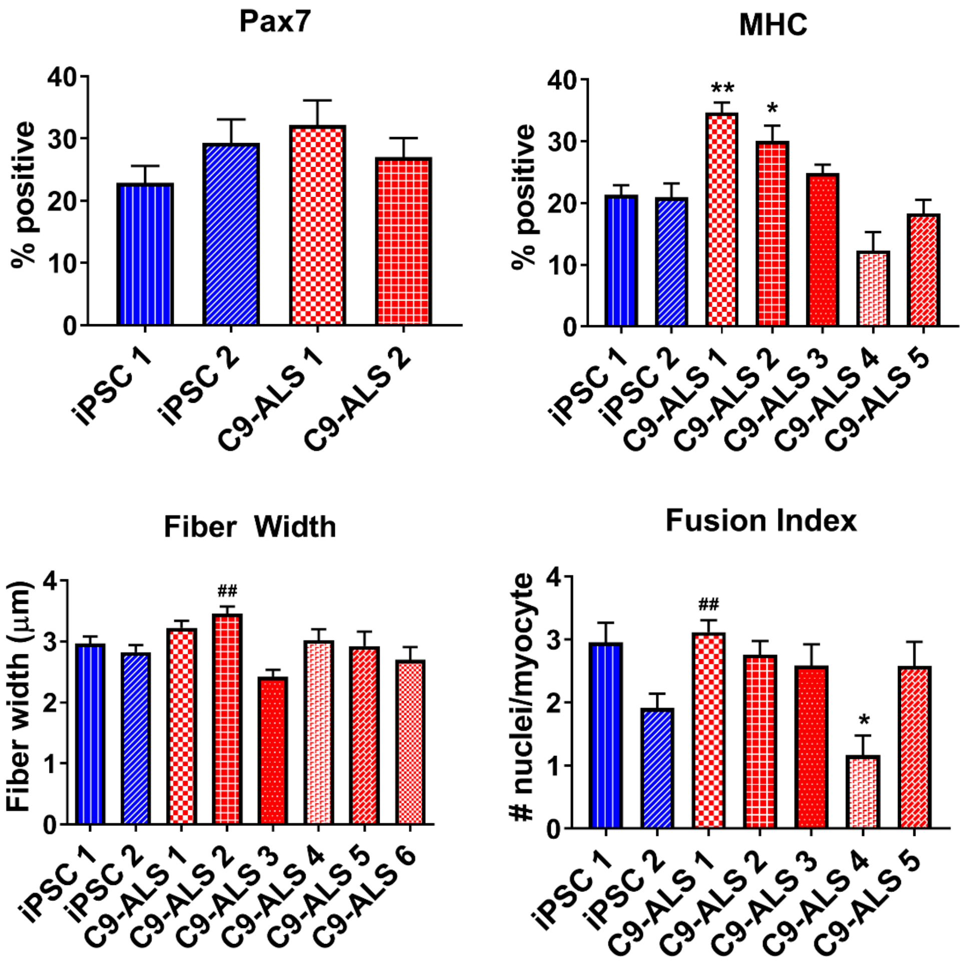

Fig. S1. Data from individual cell lines that were used to find the averaged values in **Fig. 1C-E**. Error bars represent s.e.m. from at least six fields of view. One-way ANOVA and Tukey's multiple comparisons post-hoc test were used to determine statistical significance. MHC: *P<0.05; **P<0.01 compared to both control lines. Fiber Width: ##P<0.01 compared to iPSC 2 only. Fusion Index: ##P<0.01 compared to iPSC 2 only; *P<0.05 compared to iPSC 1 only.

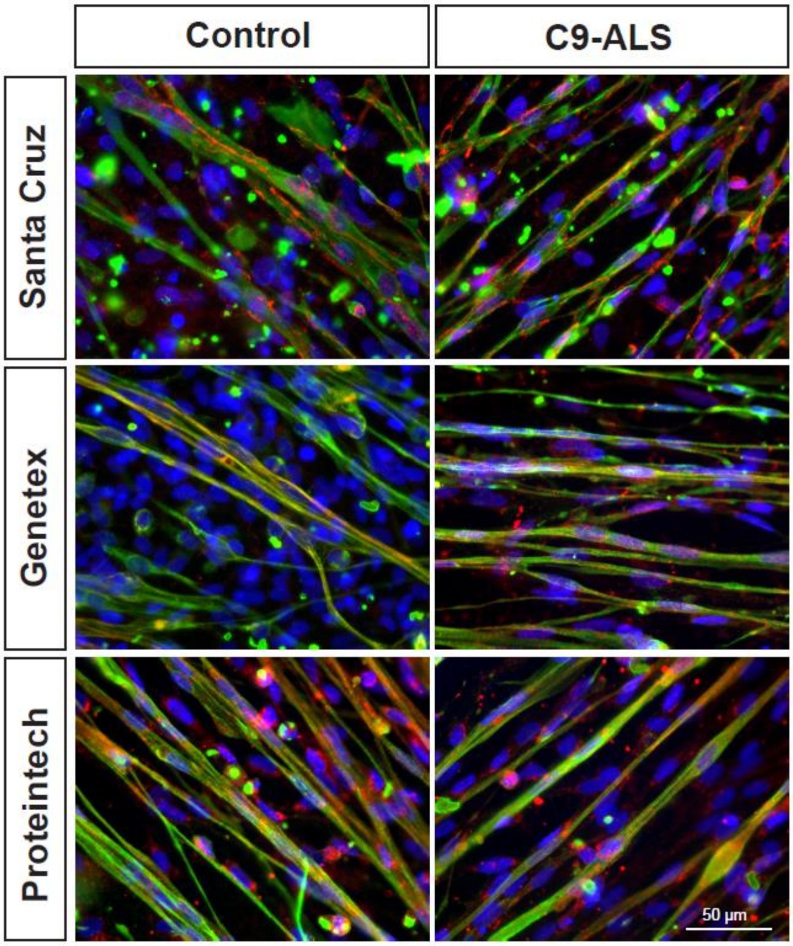

Fig. S2. Representative images for comparison of three different anti-C9orf72 antibodies (shown in red): Santa Cruz Sc-138763, Proteintech 22637-1-AP, and Genetex GTX119776. MHC is shown in green, Hoechst in blue.

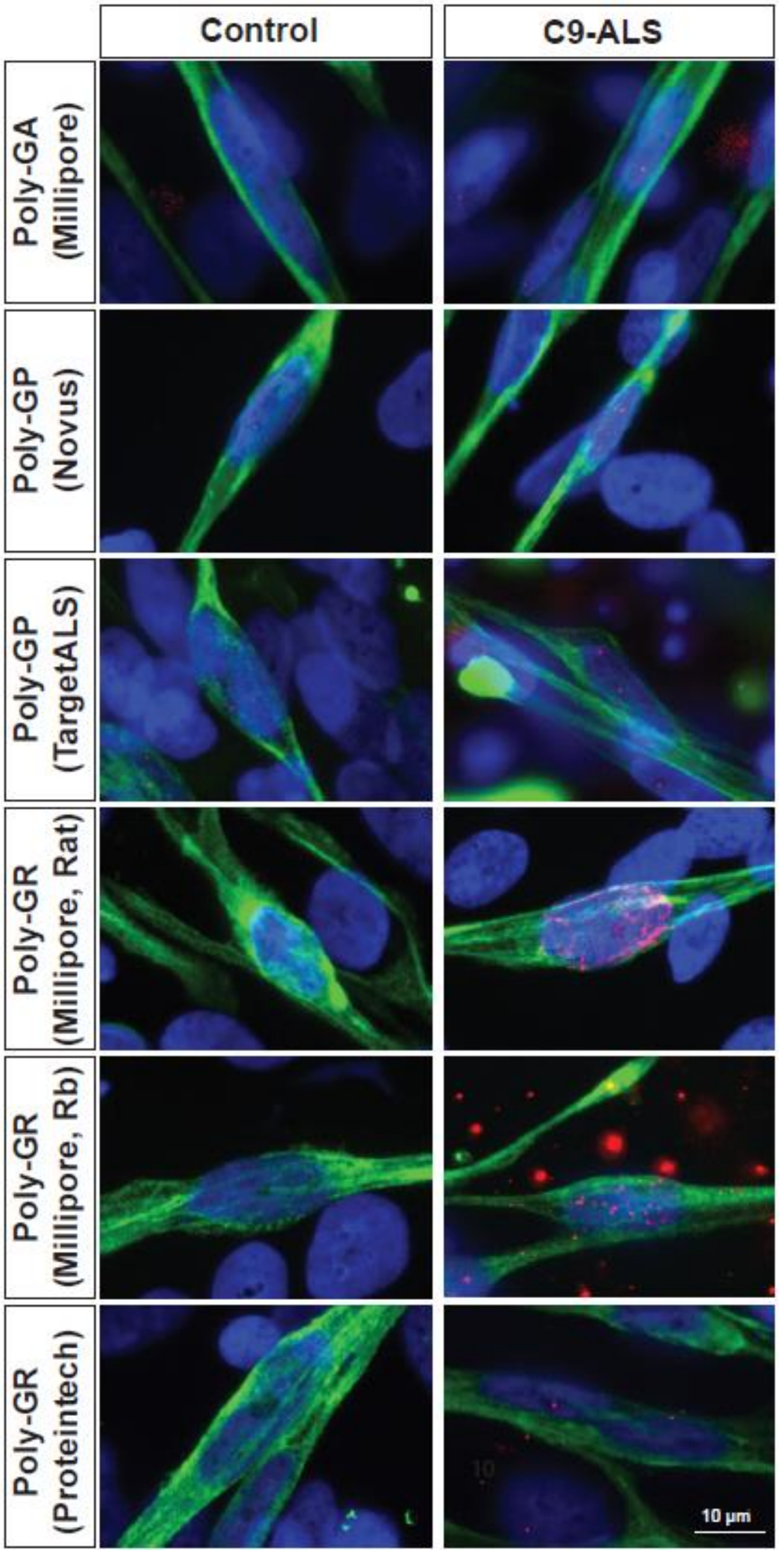

Fig. S3. Representative images for comparison of the six different antibodies against DPR proteins (details listed in **Supplemental Table 1**). Myosin heavy chain is stained in green; each DPR antibody in red, and blue is Hoechst.

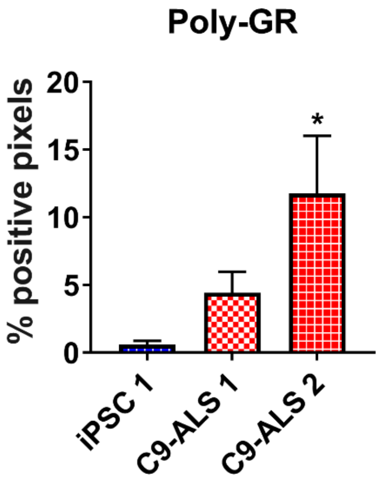

Fig. S4. Quantification of poly-GR expression at the 3-week time point for one representative set of immunostaining. Six images were taken for each line and ImageJ plug in Color Pixel Counter was used to generate the percentage of positive pixels in each image. The data was statistically analyzed by one-way ANOVA with Tukey's multiple comparisons post-hoc test.
*P<0.05 vs. iPSC1).

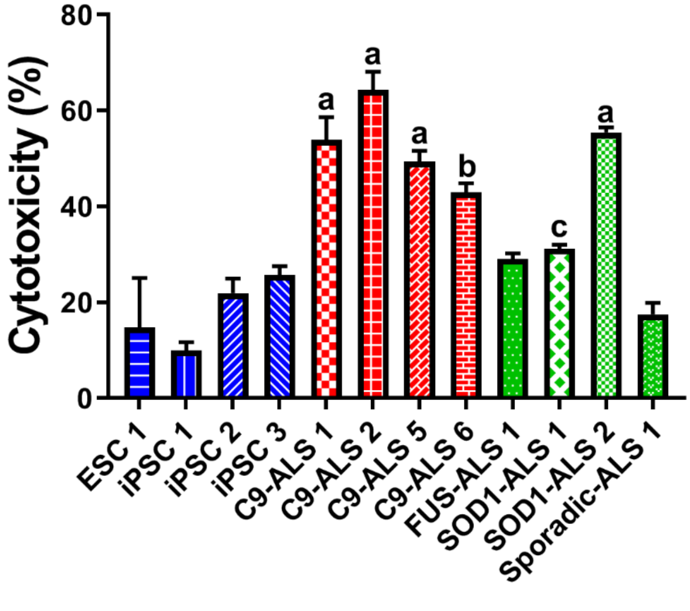

Fig. S5. The toxicity study from **Fig. 4F** was conducted on several additional ALS patient iPSC lines, including two *SOD1* lines: "SOD1-ALS 1" (N193K mutation, from NIH/NINDS repository) and "SOD1-ALS 2" (A4V mutation, from Target ALS ID TALSSOD1A4V-39.7), one *FUS* line (from NIH/NINDS repository), and one sporadic line (Target ALS ID TALSSPO21.21). One-way ANOVA with Tukey's multiple comparisons post-hoc test was performed to identify statistical significance (P<0.05). a: P<0.05 compared to all four controls; b: significant compared to all controls except iPSC 3; c: significant only compared to iPSC 1 control.

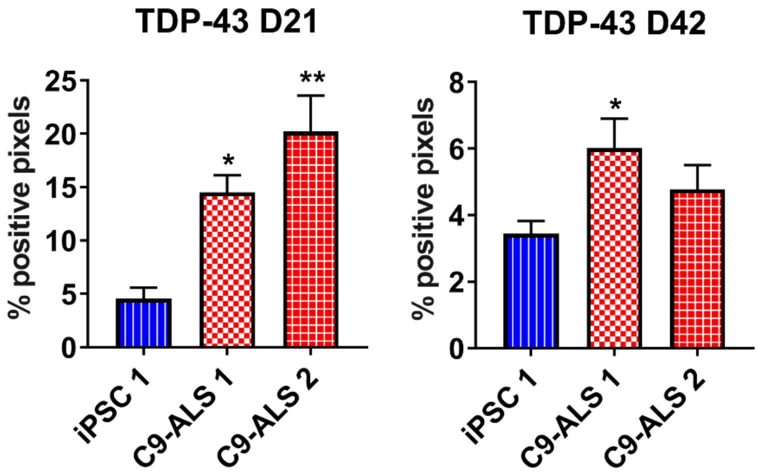

Fig. S6. Quantification of TDP-43 expression at the 3-week time point for one representative set of immunostaining. Six images were taken for each cell line and Image J plugin Color Pixel Counter was used to generate the percentage of positive pixels in each image. One-way ANOVA followed by Tukey's multiple comparisons post-hoc test was used for statistical evaluation. *P<0.05 and **P<0.01 vs. iPSC1.

Supplemental Movies

Supplemental movie files may also be viewed at the following Box link:
https://uwmadison.box.com/s/1wi1wy1nrtltp4tl6fky7yql6i3wudi7

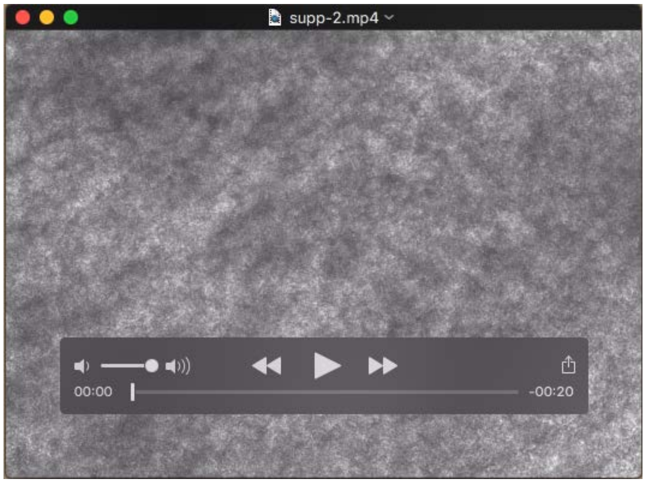

Movie 1. Control line "iPSC 1" spontaneously contracting at Day 40 of terminal differentiation.

Disease Models & Mechanisms • Supplementary information

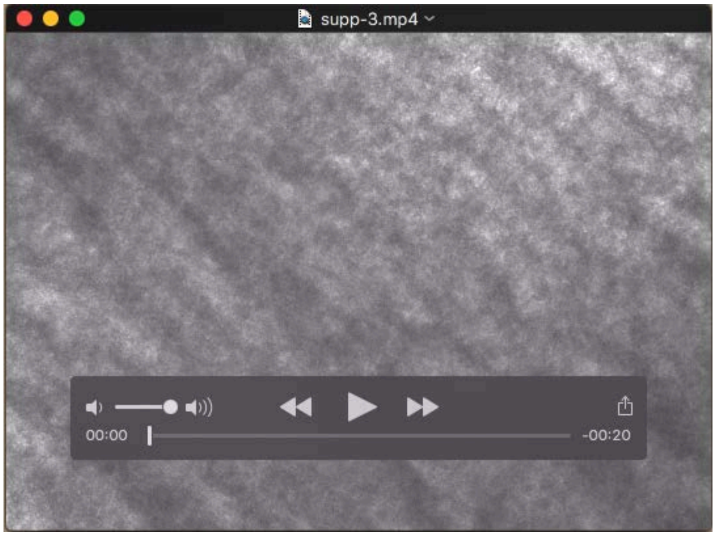

Movie 2. C9-ALS line "C9-ALS 1" spontaneously contracting at Day 40 of terminal differentiation.

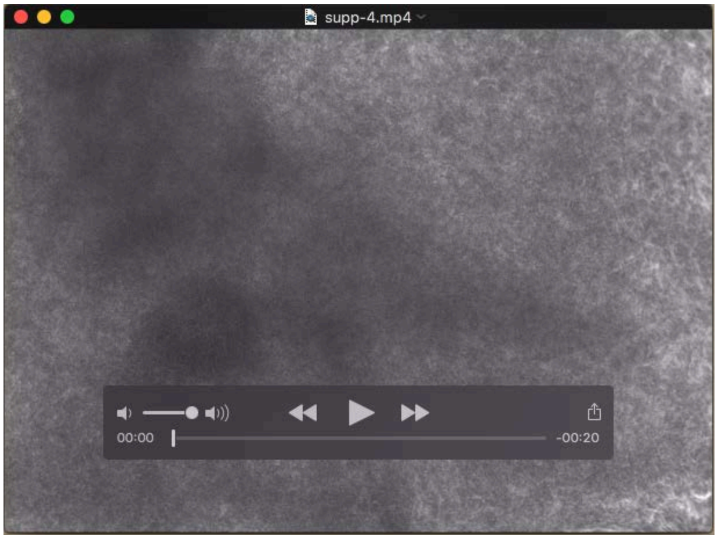

Movie 3. Control line "iPSC 1" contractions stimulated by 10 mM caffeine at Day 28 of terminal differentiation.

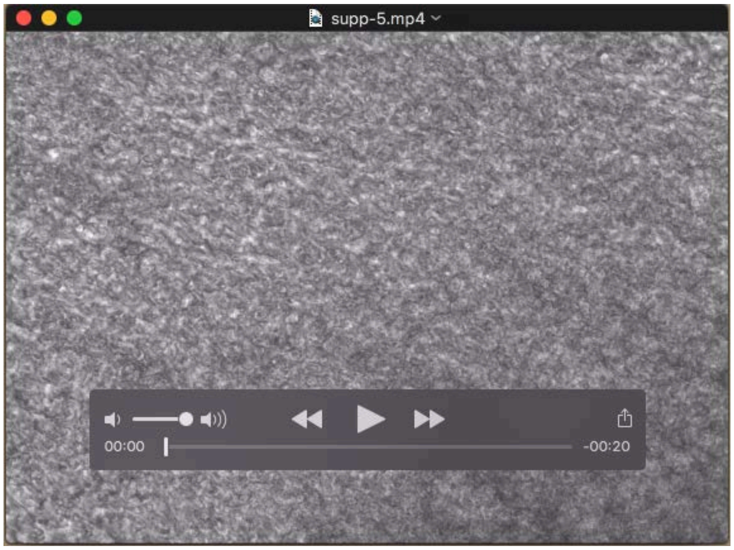

Movie 4. C9-ALS line "C9-ALS 1" contractions stimulated by 10 mM caffeine at Day 28 of terminal differentiation.

Disease Models & Mechanisms • Supplementary information

Gene	Catalog #	Vendor	Host Species	Dilution
Pax7		Developmental Studies Hybridoma Bank (DSHB)	Mouse	1:40
MyoD	554130	BD Biosciences	Mouse	1:40
MyoG	F5D	DSHB	Mouse	1:40
MHC	MF-20-c	DSHB	Mouse	1:40
MHC, conjugated to Alexa Fluor 488	53-6503-80	eBioscience	Mouse	1:500
Titin	9 D10-c	DSHB	Mouse	1:40
C9-RANT (pooled GA, GP, GR)	NBP2-25018	Novus Bio	Rabbit	1:2000
Poly-GA DPR	MABN889	Millipore	Mouse	1:500
Poly-GR DPR	MABN778	Millipore	Rat	1:2000
Poly-GR DPR	23978-1-AP	ProteinTech	Rabbit	1:500
Poly-GR DPR	ABN1361	Millipore	Rabbit	1:5000
Poly-GP DPR	TALS 828.179	Target ALS	Mouse	1:500
C9orf72	Sc-138763	Santa Cruz	Rabbit	1:500
C9orf72	22637-1-AP	Proteintech	Rabbit	1:100
C9orf72	GTX119776	GeneTex	Rabbit	1:1000
TDP-43	10782-2-AP	ProteinTech	Rabbit	1:2000
pTDP-43	66318-1-Ig	ProteinTech	Mouse	1:500
Anti-mouse IgG conjugated with Alexa Fluor 488	A21202	Thermo Fisher	Donkey	1:1000
Anti-mouse IgG conjugated with Cy3	715-165-150	Jackson ImmunoResearch	Donkey	1:1000
Anti-rat IgG conjugated with Cy3	712-165-153	Jackson ImmunoResearch	Donkey	1:1000
Anti-rabbit IgG conjugated with Cy3	711-165-152	Jackson ImmunoResearch	Donkey	1:1000

Table S1. Catalog numbers and dilution information for the antibodies used for immunocytochemistry on the skeletal myocytes.

Gene name	Forward sequence	Reverse sequence
TARDBP	AAATGGTACTCGGGGACCTC	GAGGCAGAATTGCTTGAACC
HNRNPK	CGGGAGCTTCGATCAAAAT	TCCAGAATACTGCTTCACACTG
GPC3	TGGACAAGAACTCGTGGAGA	AGGAAAAAGTGTGCCTGGTG
DCX	ATGAAGGGAAACCCATCAGC	TACAGGTCCGTCTTGGTCGT
BET1L	AAGTCACCAGGCTCAAATCGC	GAGGAATCCCAGCAAGATCA
RPS3	CTGAAAAGGTGGCCACTAGA	CCCACTCTCCATGATGAACC
SIGMAR1	GTCCGAGGGAGACGGTAGTA	GAAGTCCTGGGTGCTGAAGA
RHOU	CTGCCTTCGACAACTTCTCC	AGAGGCCTCAGCTTGTCAAA
TIMM9	TAGGGACGTGGGATTAGGTG	GTCCCCAGAAATTCCTTAAAC
ATP5A1	TCGTGTAGTTGATGCCCTTG	TCGTGTAGTTGATGCCCTTG
NDUFB11	TGGGATGGGATGAAAGAGTG	GGGTCGAAGCAGTTGGATT
ACTB	CCAACCGCGAGAAGATGA	CCAGAGGCGTACAGGGATAG

Table S2. Primer List for RT-qPCR.

Disease Models & Mechanisms • Supplementary information
